# Why do some women choose to freebirth in the UK? An interpretative phenomenological study

**DOI:** 10.1186/s12884-016-0847-6

**Published:** 2016-03-21

**Authors:** Claire Feeley, Gill Thomson

**Affiliations:** University of Central Lancashire, School of Community and Midwifery, Flyde Road, Preston, Lancashire PR12HE UK

**Keywords:** Pregnancy, Childbirth, Freebirth, Maternity, Midwifery, Care provision

## Abstract

**Background:**

Freebirthing or unassisted birth is the active choice made by a woman to birth *without* a trained professional present, even where there is access to maternity provision. This is a radical childbirth choice, which has potential morbidity and mortality risks for mother and baby. While a number of studies have explored women’s freebirth experiences, there has been no research undertaken in the UK. The aim of this study was to explore and identify what influenced women’s decision to freebirth in a UK context.

**Methods:**

An interpretive phenomenological approach was adopted. Advertisements were posted on freebirth websites, and ten women participated in the study by completing a narrative (*n* = 9) and/or taking part in an in-depth interview (*n* = 10). Data analysis was carried out using interpretative methods informed by Heidegger and Gadamer’s hermeneutic-phenomenological concepts.

**Results:**

Three main themes emerged from the data. *Contextualising herstory* describes how the participants’ backgrounds (personal and/or childbirth related) influenced their decision making*. Diverging paths of decision making* provides more detailed insights into how and why women’s different backgrounds and experiences of childbirth and maternity care influenced their decision to freebirth. C*onverging path of decision making,* outlines the commonalities in women’s narratives in terms of how they sought to validate their decision to freebirth, such as through self-directed research, enlisting the support of others and conceptualising risk.

**Conclusion:**

The UK based midwifery philosophy of woman-centred care that tailors care to individual needs is not always carried out, leaving women to feel disillusioned, unsafe and opting out of any form of professionalised care for their births. Maternity services need to provide support for women who have experienced a previous traumatic birth. Midwives also need to help restore relationships with women, and co-create birth plans that enable women to be active agents in their birthing decisions even if they challenge normative practices. The fact that women choose to freebirth in order to create a calm, quiet birthing space that is free from clinical interruptions and that enhances the physiology of labour, should be a key consideration.

## Background

In the developed world, it has been identified that a minority of women choose to birth without the assistance of a midwife or doctor and instead choose to either birth alone or with a lay birth supporter present [1, 2]. This is known as freebirthing or unassisted childbirth. This is a different phenomenon to that of a concealed pregnancy, which is often characterised by a denial of the pregnancy [3], or when women have restricted or no access to maternity care. Freebirthing is a unique phenomenon, whereby women make an active choice not to utilise the maternity services that are available to them.

While the direct risks of freebirthing are unknown due to its covert nature, a parallel in terms of risk relates to when women give birth unintentionally without a healthcare practitioner present, known as ‘born before arrival’ (BBA). For example, a cohort study carried out by Loughney et al. [4] suggests this occurs in 0.14–0.44 % of pregnancies in the UK. BBA’s are unplanned and associated with an increased morbidity for mother (excessive blood loss) or baby (failure to retain body temperature), although overall outcomes are normally good [4]. The World Health Organisation (WHO) strongly advocates that all women and babies need skilled care in pregnancy, childbirth and immediately after [5]. WHO estimate that 10–15 % of pregnancies and/or birth will have obstetric complications needing intervention for optimal outcomes [5]. The types and prevalence of risks during labour include, obstructed labour (incidence 8 %), [6], pre-eclampsia (incidence 2–8 %) [7] post-partum haemorrhage (incidence varies dependent upon risk factors) [8], shoulder dystocia (incidence 0.5 %), [9], neonatal encephalopy (incidence variable and the statistics are unclear), [10] and cord prolapse (incidence 0.1–0.6 %) [11]. Often these risks are unforeseen and require timely intervention for good outcomes [5, 12]. These insights thereby highlight that the freebirthing woman (and her baby) has a potential increased risk of morbidity or mortality.

A recent metasynthesis [13] was undertaken to explore *‘Why do some women choose to freebirth?*’ While only four primary studies were identified; three of which were undertaken in the US [14–16] and one in Australia [17], they included narratives of 272 women. Four key themes were generated; rejection of the medical *and* midwifery models of birth; faith in the birth process; autonomy; and agency (the ability to make and carry out own decisions). While methodological weaknesses were identified in the studies, the findings offer important insights into women’s mistrust of any form of maternity care and their faith in the physiology of an undisturbed birth. Furthermore, there was a prevailing sense of choosing to freebirth in order to retain choice, control and autonomy over their bodies during the birth process [13].

One of the recommendations of the meta-synthesis was to explore the motivations and decision making of women who chose to freebirth in the UK. To date there has been no primary studies undertaken in this context despite anecdotal evidence of its occurrence [2, 18–21]. Maternity services in the UK are very different to that of the US and Australia. The UK has a National Health Service, whereby maternity care is free for all pregnant women providing they meet the criteria of residing in the UK for >1 year, otherwise known as ‘ordinarily resident’ [22]. In the UK, unlike US and Australia, midwifery-led care is the default framework in which women access care. Consequently, the findings of the meta-synthesis cannot be transferrable to other settings, leaving a gap in terms of understanding this phenomenon and its occurrence from a UK perspective. Given the potential risks to mother and baby, it is an important topic to explore further. Furthermore, the findings will be of benefit to maternity professionals in terms of identifying women’s childbirth needs, how to meet these needs and to illuminate any potential shortfalls in current provision.

The aim of this study was to identify and explore what influenced women’s decision to freebirth in a UK context.

## Methods

### Design

An interpretative (hermeneutic) phenomenological approach underpinned the study design. Phenomenology seeks to illuminate the lived experience of a shared phenomenon [23]. Emphasis is placed on the subjective experience of the participant and the meanings they attribute to their experience, thereby allowing the researcher to gain insights into people’s motivations and actions [24]. It integrates the relationship between socialisation, enculturation and how we interpret our lifeworld [25]. Therefore, our interpretations, or the meanings we place upon a phenomenon are constructed within a socio-cultural context [26]. This approach also identifies how understanding is a matter of negotiation between the researcher and those being researched; with the insights generated being co-constructed within an interpretive dialogue. Rather than the researcher ‘bracket’ (i.e. set aside) their pre-understandings about the phenomenon in question, the researcher makes explicit their pre-suppositions and through reflexivity ensures that they do not mask or inhibit new insights from the data [27].

### Reflexivity

To fulfil the reflexivity aspects of an interpretative phenomenological design, the lead author (CF) was interviewed by the second author, GT. This was prior to any data collection taking place to make explicit any pre-suppositions. CF did not transcribe the interview but listened to it several times, documenting key points. The interview highlighted that CF is a midwife with a firm philosophy of care embedded within a woman-centred approach. However, and due to limitations of current maternity practices, she felt that this ethos of care is not always realistic or attainable. This explication of pre-understandings and potential ‘biases’ was used to foster an open and authentic basis that was referred back to and reflected upon throughout the study. A reflective journal was also used by CF for initial thoughts and interpretive ideas to be recorded following each interview and throughout the analysis process.

It was recognised that being a midwife-researcher could have been a potential barrier for the participants to discuss their motivations, without fear of condemnation. To overcome this, CF shared her philosophy of care and the aims of the study with the participants prior to data collection.

### Recruitment and participants

A purposive sampling method was used via social media freebirth support groups. Permission to advertise the study was sought by the website owners and once granted an advertisement was posted. A snowball technique was also used whereby participants were asked to forward information to anyone else who matched the inclusion criteria. Women, who were over 18 years old, had intentionally carried out a freebirth in the UK and who were English speaking were eligible for inclusion. The recruitment phase took place over a two week period in September 2014.

Women who were interested in the study were invited to make initial contact by email. An information sheet with explicit information regarding the study aims, participatory requirements, voluntary nature of participation and confidentiality was then issued. All interested participants were asked to confirm whether they were still willing to take part, following which a consent form and further details regarding participation (i.e. a narrative guide and instructions for password protecting documents) were provided. Consent for participation and dissemination of the findings was gained in two stages, prior to the completion of the narrative and prior to the interview. It was noted that the participant’s geographical location varied widely, indicating that this phenomenon may occur anywhere in the UK.

### Data collection

There were two primary methods of data collection; narratives and telephone interviews. While interviewing is accepted to be the main source of data collection for interpretative phenomenology, Bamberg [25] discusses how the narrative form can provide a portal into the participant’s realm of experience. Furthermore, narrative as a method, can help participants with personal ‘sense-making’ of an experience [25], as well as bring forward the participants first stage of interpretation, which aligns itself well with interpretative phenomenology.

The participants were invited to provide a narrative of any length prior to an interview being undertaken. The aim of the narrative was for the participant to provide an account of their decision to freebirth in an unstructured way, although prompt questions were included in a narrative guide to assist the process. Once the narrative was completed, the participant was asked to forward it to the lead author via a password protected email.

The majority of interviews were carried out over telephone or Skype. In one occasion the participant preferred an online instant messaging format whereby an encrypted chat room was used (https://www.svyft.com/). The interviews were audio-recorded, took between 30 min and 2 h to complete and were transcribed in full. A semi-structured interview style was adopted in which the participants were invited to further discuss the narrative information provided. The questions were individualised for each participant, and were primarily open ended questions to encourage further dialogue based upon the narrative provided. Questions such as, ‘Can you tell me more about X?’ ‘How did you feel when X happened?’ ‘What did you mean by X?’ were commonly used. In the event that a narrative was not provided, a prompt sheet of questions was created based upon the narrative guide that the participants were issued with. A ‘conversational’ manner was adopted during the interview in which the lead author shared her personal and professional interest in the topic. This approach aimed to provide the safe and trusting space as described by Laverty [28] and to encourage the participants to share detailed accounts of their experiences. Demographical information was also collected with consent. Following the final synthesis, member checking was facilitated by sending the participants a copy of the findings with the opportunity to comment. Six participant’s responded and all provided positive feedback that the findings reflected their views.

### Data analysis

Heidegger’s and Gadamer’s interpretative phenomenological concepts guided data analysis; whereby the hermeneutic circle offers a theory and methodology for analysis [24]. This represents an iterative process, where the individual meaning parts of the texts/transcripts are viewed in context of the whole narrative, and the whole is understood by the cumulative meanings of the individual parts [26].

Interviews were transcribed by CF to allow immersion in the data and to assist the analysis process and all data was uploaded to MAXQDA [29], a qualitative software data management programme. Each narrative and interview was analysed line by line using an ‘in-vivo’ method whereby poignant descriptive phrases illuminating key concepts pertinent to the research question were highlighted and assigned a code [30]. This was continued until saturation was reached and no new codes emerged. All codes were then reviewed iteratively and the dominant codes formed the basis of initial tentative themes and allowed disconfirming data to emerge.

The themes were further refined and developed through the process of writing as endorsed by Van Manen [23]. He considered writing to be an integral part of the analysis and essential to the interpretative process whereby the researcher can work and re-work the emerging themes. This iterative process of coding, creating themes and re-writing continued until coherent interpretations emerged.

All analytical decisions were discussed and shared with GT which contributed to a more coherent and structured ‘story’ to illuminate the research question.

### Ethics

Ethical approval was obtained from the Science, Technology, Engineering, Medicine and Health (STEMH) Ethics Committee at the University of Central Lancashire in June 2014, and an amendment was approved in January 2015 (project number: STEMH 208).

### Results

#### Participant characteristics

Ten women were consented to the study. Nine women completed a narrative prior to the interview and 10 women took part in an interview. Participant demographics and birth data were collected (refer to Table 1). These data highlight variations in the participants’ age and parity, although the majority were from a Caucasian ethnic background. All of the women indicated a high level of education ranging from A-Levels to Postgraduate training and all were either married/living with partners at the time of the interview.

**Table 1:** Participant characteristics

Demographic information	*N* (%)
Age (years)	
25–29	1 (10.0 %)
30–34	3 (30.0 %)
35–39	4 (40.0 %)
40+ years	2 (20.0 %)
Ethnicity:	
White British	6 (60.0 %)
White Other	3 (30.0 %)
Mixed Race	1 (10.0 %)
Marital Status	
Married	7 (70.0 %)
Living with partner	3 (30.0 %)
Highest educational level	
A-Levels/Equivalent	1 (10.0 %)
Diploma/Higher Education Cert	3 (30.0 %)
Degree	5 (50.0 %)
Postgraduate	1 (10.0 %)
Employment status	
Stay at home mother	3 (30.0 %)
P/T employed/study	4 (40.0 %)
F/T employed/study	3 (30.0 %)
Number of children	
One	1 (10.0 %)
Two	5 (50.0 %)
Three	1 (10.0 %)
Four+	3 (30.0 %)
Range of freebirths	
One	6 (60.0 %)
Two	3 (30.0 %)
Three	1 (10.0 %)

Overall these women had a collective total of 33 birth experiences, 31 were normal vaginal births and two were assisted births. Eleven of the births took place in hospital, one in a birth centre, six were homebirths and 15 were freebirths. All freebirth experiences took place between 2006 and 2014. None of the women experienced a perinatal loss.

Three key themes that explored women’s motivations to freebirth emerged from the data set: ‘*contextualising herstory’*; ‘*diverging paths of decision making’* and ‘*the converging path of decision making’*. These themes describe what and how underlying factors or previous life experiences led the women to freebirth, as well as highlighting diverging and converging influences on how and why these decisions were made. A pseudonym for the participants has been used to ensure anonymity, together with the data source i.e. narrative or interview and associated line numbers from the transcripts.

### Contextualising ‘herstory’

This first theme highlights the different contexts of the participants ‘herstories’ – a feminist reclamation of history, from the female perspective [31] – which they felt framed their decision to freebirth. The sub-themes of ‘*personal herstories’*, ‘*inherited birth beliefs’* and *‘embodied birth experiences’* explore different aspects of their stories. Whilst generalisations cannot be made about *how* underlying factors and life experiences shaped the participants worldview for its impact is felt in a unique way for each woman, these insights framed how their decisions during childbirth were formed. These self-reflections provided a sense of understanding of the individual nature of each participant’s life at the point prior to their decision to freebirth: unique and different but all with a sense that where they began was relevant to their subsequent journey.

#### Personal herstories

Within each narrative the participants’ disclosures about their lives prior to freebirthing provided a rich contextual backdrop which framed their stories and that of their decision to freebirth. One woman had been raped and three reported enduring abusive relationships which had left them recovering from mental health disorders such as ‘*PTSD*’ and *‘anxiety’*. One participant described how her abusive childhood had impacted on her world view:

*‘I absolutely hate to feel helpless, lied to or pushed around by people who think they are smarter/better than me, because of this.’* (Holly, nar, In: 4–4).

Other women described certain aspects of their lives which they felt were pertinent to provide the context as to why they chose to freebirth. These included a participant who had a diagnosis of Asperger’s Syndrome and *‘situational autism’*. Another participant had a difficult childhood marked by a father leaving the family home which left the participant with a need for *‘self-reliance and control’.* One referred to how she had *‘low self-esteem’* due to range of negative life events and another reported that she did not have any support around her prior to pregnancy and childbirth.

#### Inherited birth beliefs

In contrast to the difficulties that many of the participants experienced during their lives, four women described how their family herstory of homebirth created a sense that birth was a *‘normal part of life’,* yet *‘special’*. For these participants a homebirth was described as an ‘*idealised*’ life event, and informed a part of their herstories in which they enjoyed their mother’s recounting their birth stories:

*‘I myself was born at home, with a midwife and to me that was idealised, a homebirth was something that has pleasant memories for me well pleasant nostalgia because my mum said ‘oh you were born at home’, you know, ‘I was walking around hanging out the laundry the day before and the next day, I couldn’t believe it I had a baby that night!’ That birth story, wasn’t so much that it was great, it was just normal.’* (Cat, int In: 23–23).

This positive inherited social enculturation as well as the subconscious memory of being born at home contributed to how the women framed birth and freebirth within their world view:

*‘Yea, I remember the way she spoke, you know the way with body language and things, the way she spoke about her homebirths, yes there were a few stressful things, but there was humour and she was relaxed and things, but the way she spoke about it was very much like the freebirthing women spoke about their births.’* (June, int, In: 9–9).

#### Embodied birth experiences

All of the participants bar one had at least one previous childbirth experience prior to making a choice to freebirth. These women reported a diverse spectrum of positive and negative childbirth experiences, however, all of them had had a negative experience of maternity care in some capacity. These experiences ranged from being *‘irritated’* by the midwives hindering their birthing experience, to feeling that their expectations had not been met. These latter occasions were when the midwives assumed more of a ‘*medical role’* as opposed to a ‘*mothering role’* expectation:

*‘The only thing was that I’d had to work quite hard to feel undisturbed by the midwives and the busy, bossy vibe they had added to our birthing environment. X (husband) had to remind them several times that I wanted complete silence, as they would chat about holidays just outside the door.’* (Jenny, int, In: 4–4).

Six of the women also provided self-reports of a *‘traumatic birth’*:

*‘I felt violated and humiliated. It ended up with the doctor telling me my baby was stuck and she would try to pull my baby out, in theatre, with an epidural, surrounded by strangers, in case it didn’t work in which case they would perform an emergency c-section. It was the most awful experience of my life.’ (*Jane, nar, In:2–2).

All occasions of birth trauma occurred during a hospital birth. Repeated incidents of women *‘being ignored’*, *‘left alone’* or conversely *‘harassed’* by hospital midwives and doctors left the women feeling *‘abandoned’*, *‘disempowered’*, *‘out of control’* and ‘*frightened’*. Non-consensual acts were carried out including vaginal examinations and IV lines being inserted where the women reported being *‘done to*’, rather than being a part of an informed process. These experiences often evoked ‘*shame’* wherein the women seemed to internalise the actions of the maternity staff and blamed themselves for not stopping them:

*‘I felt ashamed, the only other thing I have ever felt ashamed of uh through the whole process, I was ashamed that I sounded like a pig that’s being slaughtered.*’ (Cat, int, In: 25–25).

*‘And looking back I was like why did I consent to having syntocinon with a baby that could potentially could have been distressed? It didn’t make sense.’* (Kate, int, In: 23–23).

In contrast, seven of the participants described positive experiences of birth, all of which had taken place in non-obstetric settings i.e. two of the women gave birth at a birth centre, and five had planned homebirths with midwives present. The adjectives used to describe the birthing experience included: *‘wonderful’*, *‘calm’*, *‘perfect’*, *‘easy’* and *‘beautiful’*. Of interest, was the participant’s relationship to their midwives, where their ‘*quiet presence’* who seemed to be not *‘doing much’* was highly valued by the women. Others reported feeling supported by their midwives, which in turn helped to facilitate a positive birth experience. These narratives provided a stark contrast to the other women’s negative experiences as they were able to reap the benefits of a calm atmosphere and supportive but quiet midwives who simply ‘*let them get on with it’*.

### Diverging paths of decision making

This theme provides more detailed insights into how and why women’s different backgrounds and experiences of childbirth and maternity care influenced their decision to freebirth. The subthemes of *‘instinctive’*; ‘*compounding trauma’* ‘*seeking solace in homebirth’*; and *‘improving and enhancing the birth experience’* explore these different paths in more depth.

#### Instinctive

For one participant, the path to freebirthing was entirely instinctual. Claire had no prior experience of birth but had made the decision to freebirth during her first trimester in pregnancy. Claire had been proactive from the start of her pregnancy in seeking out her birth options as she knew immediately that she would not birth in hospital. It was during her research into birth options that she came across the concept of freebirthing and instantly knew it was the right decision for her:

*‘I hadn’t really explicitly thought about where/how to give birth before then, but if I had, I would have identified immediately that it wouldn’t be in hospital, and I didn’t want anyone else around. So as soon as I came across the concept* [freebirthing]*, it made complete sense to me.’* (int, In: 4–4).

This belief in part stemmed from her self-awareness of her personal needs in which she identified as *‘not naturally sociable’* and an inner knowing that having midwives around would cause her *‘stress’* which she believed *‘would make things more likely to go wrong, not less.*’ For Claire, there was no distinction between midwifery or medical care, and she rejected both models of care. It would seem that freebirthing was the only option that she deemed suitable for her needs. Thus, her decision to freebirth was a remarkably straightforward one.

#### Compounding trauma

In an attempt to overcome a previous traumatic birth, three participants booked a homebirth in their next pregnancy. Unfortunately, these women (Julie, Holly and Cat), experienced negative interactions with their community midwives. This compounded their previous trauma which in turn led them all to change their birth decision to freebirth. These women felt that again they *‘weren’t being listened to’* and that they were being *‘manipulated’* and *‘bullied’* for making informed decisions to book their homebirth*.* They likened these negative interactions as *‘going into battle’* at routine antenatal appointments which they found *‘stressful’*. These women felt immense pressure to comply with local policies and guidelines and were referred to Consultant Obstetricians when they did not comply. They perceived that their care did not consider their individualised needs, knowledge or preferences for birth:

*‘I felt no faith whatsoever in my local maternity service in 2006. No trust. No support. Nothing but revulsion for their attitudes and revolving door policies, and for the lies and pressure they put me under without understanding I am a smart and educated girl.’* (Holly, nar, In: 5–5).

These women also reported that their community midwives seemed to be ‘*fearful of birth’,* which in turn eroded their confidence in being attended by them during their home birth. Furthermore, an awareness that it was a *‘lottery’* as to who attended their homebirth, meant that they did not want to take the risk of having a fearful or unsupportive midwife look after them in labour:

*‘The obstructive behaviour by the community midwives, the lottery of who would turn up at the birth. If their behaviour was indicative of many of the midwives in the Trust then I could not trust that they were supportive of home births. I actually became fearful that they would turn up in time for the birth as they seemed more scared of attending a home birth than I felt about having a home birth.’* (Cat, nar, In: 8–8).

For Cat, this experience with the midwives coupled with an increasing sense that midwives would *‘block her birth’* made a decision to freebirth at 30 weeks gestation. Julie also booked a homebirth, but during her labour she decided she did not want midwives there as she felt they would interrupt and intrude upon the *‘safe haven’* of her birthing space. For these women, safety did not mean midwifery attendance, rather they felt that midwives would have hindered the birth process through jeopardising their feelings of safety and security. Lack of trust in the service provision was a prevailing feeling. In this respect, the unsupportive and at times obstructive behaviour of the NHS midwives facilitated the decision to freebirth.

#### Seeking solace in homebirth

Three of the women who had a traumatic birth went onto have at least one successful homebirth with community midwives in attendance before they carried out a freebirth. For these women, they knew that they wanted to freebirth but lacked *‘faith’* in themselves. Within their homebirth accounts, there was a sense that the women sought the support of midwives in order to ‘*prove’* that their bodies could birth safely, a confidence that had been eroded by their traumatic experience:

*‘I think in hindsight I probably needed to prove to myself I was capable of doing it before contemplating doing it alone.’* (June, nar, In: 8–8).

By seeking solace in homebirth these women described a great sense of *‘empowerment’* and indicated that it was an affirmation of womanhood. Their accounts of their interactions with midwives were in stark contrast to those they had previously experienced. They reported that the community midwives were very supportive of their decision to homebirth and even in two cases, implied support for a freebirth. These women valued the community midwives support, feeling that they were *‘listened to’* and thus consequently that their individual needs for birth were valued. In particular, a midwife who came across as *‘hands off’* and who was an advocate for the woman was appreciated:

*’My second a beautiful homebirth, luckily supported by a case loading team in X (Trust). The NHS care I received from midwives was outstanding and I wrote a letter of commendation.’* (Kate, nar, In: 4–4).

#### Improving and enhancing the birth experience

Three of the women had only had positive experiences of childbirth (two had homebirths, one had a hospital birth) prior to making a decision to freebirth.

The women described how they had a firm sense that their body *‘could do it’*, due to *‘trust’* in their inherent capabilities and their ability to tune into their *‘instincts’* during birth:

*‘I already knew from the first birth that when I have space to internalise, to tune into that super strong survival instinct a birthing mother has, that I know whether all is well, or not.*’ (Jenny, nar, In: 5–5).

While the midwifery care they had previously received was positive, the women still held a sense of how their presence had detracted them from *‘tuning in’* to their physiological responses, and *‘disturbed’* their birth space:

*‘Well the fascinating thing is that because the midwife was talking to me regularly during contractions, I was very irritated by her presence (laughs).*’ (Nicky, pn-10, int, In: 11–11).

Their motivation to freebirth evolved when considering their needs during their future birth. Women talked about how they wanted to achieve *‘greater depth to the experience’.* This involved a rejection of medical involvement wherein their trust in their healthy pregnancies and ability to give birth meant that they deemed *‘monitoring, checks, questions, and procedures’* as unnecessary. It also involved consideration on the role of the midwife and how they may contribute or potentially detract from their birthing experience. Knowledge of midwifery obligations and professional accountability also factored into the women’s decision making:

*‘So for the second birth, we explored ways of avoiding the disturbance, while having the safety net of a midwife present. I imagined we might ask her to stay downstairs unless I asked for her. We worried about whether this would be respected, since midwives have a job to do. We then defined for ourselves what we wanted a midwife’s role to be at this birth – it would be worst case scenario: to help identify a problem, and call for a transfer.’* (Jenny, nar, In: 5–5).

These women were keen to stress the emergence of this decision as *‘a well thought out process’*, one that took time and *‘intelligent reasoning’*:

*‘I do not believe that freebirth is a choice for everyone and it is something that I worked towards, rather than made hard, fast decisions about but I think it is crucial to stress that my choices were born out of positivity, a deep understanding of myself and intelligent reasoning.’* (Alex, pn-8, nar, In: 11–11).

### The converging path of decision making

This theme explores how the women’s paths converged as they attempted to validate their decision to freebirth. Four sub-themes are discussed in relation to: *‘understanding the physiology’*; *‘creating a protective, safe space’*; *‘creating and accessing wider support networks’*; and ‘*conceptualising risk’*.

#### Understanding the physiology

All the women reported undertaking extensive research:

*‘I researched everything. How to DIY, how to deal with the cord, my rights, EVERYTHING.’* (Holly, nar, In: 10–10).

This involved making sense of their previous birth experiences as well as research into birth physiology:

*‘I knew that with my second, I was searching for a greater depth to the experience, something more intuitive as I had come to trust myself more than I had previously, not just through experience, but through research that supported my beliefs in understanding the science behind mammalian instinct, physiological birth and the huge value of the hormonal and emotional process.’* (Alex, int, In 5–5).

Participants often referred to how knowledge of mammalian biology, where birthing alone was a normal life event, enabled them to have trust in their bodies:

*‘I accepted that like any other mammal, I can give birth so the implicit trust I have in my biology played a fundamental role in this acceptance of birthing alone.*’ (Cat, nar, In: 9–9).

This research confirmed the importance of them being *‘undisturbed’* and feeling *‘safe’* during labour as well as how a midwife would *‘medicalise’* the process through clinical check and *‘interfering’* in the natural flow of birth:

*‘And I really felt, that their presence would actually um be counter to what I believe should happen ummm and I felt that why am I actually inviting a midwife? I really thought about that.’* (Jenny, int, In: 29–29)

#### Creating a protective, safe space

For the majority of women, their decision to freebirth was made in conjunction with their partners. Women referred to how they had looked to their husbands to provide a *‘protective’* and *‘safe space’* for them to birth safely and trusted that they would action any emergency should it arise:

*‘Having gone through the wonderful homebirth together I knew that I could give birth normally and that I could trust my husband to protect and support us through the labour. He was also comfortable with things, now knowing what he needed to do and what would happen. I opted with this pregnancy to use maternity care at a minimum.’* (June, nar, In: 9–9).

As identified previously, the thought of having an unknown midwife, a ‘*stranger’* was considered unacceptable:

*‘I wondered if the midwives were contacted, but kind of knew they weren’t. I really didn’t want to change the safe haven bubble and trusted people surrounding me. I also did not want to be interfered with, examined or questioned by people I did not know.’* (Julie, nar, In: 49–49).

The women’s insights seemed to suggest that *‘trust*’ in who was present was essential.

#### Creating and accessing wider support networks

All of the participants accessed freebirthing websites and forums. These forums were described as a ‘*safe place’* to receive *‘non-judgemental’* support through *‘women supporting women’*:

*‘Throughout my preparation, I found an online Freebirth community in which I became and remain active within. It is a wonderfully complex and diverse population. I have found it to be a very open, supportive, nurturing community which holds space for women from all kinds of spheres and who go on to experience many different birth outcomes.’* (Alex, nar, In: 11–11).

Five women employed a doula during their pregnancy, to *‘help them process their feelings’* as well as provide them with a *‘wider community for support’* for this alternative birth choice. Three of the women also employed independent midwives for antenatal and postnatal care, but they were not in attendance at the birth. While these professionals did not advocate freebirthing, the fact that they provided *‘individualised care’* and supported autonomous decision-making led them to feel that they plans were respected:

*‘…um she works on a relationship with me and offered me information regardless of whatever policies. Because she didn’t have any policies, she was employed by me and providing a service to me. So the information was a lot easier to get hold of and she was a lot easier to talk to. But yea, we were able to talk to her no problem about freebirth.’* (Kate, int, In:63–63).

#### Conceptualising risk

Underpinning the women’s concept of risk was the belief that *‘interference was risky’.* Through their research they weighed up their own personal risk factors and concluded that freebirthing was their safer option:

*‘In the end, it was a risk assessment. We weighed up the likelihood of all the risks that mattered to us, and made a decision based on our level of comfort with each of those risks.’* (Jenny, pn-9, nar, 8–*8).*

The women were also concerned about being attended to by professionals whose practice did not support optimal birth physiology; *‘I found a lot of the methods used in hospitals so out of touch with natural birth it really angered me’*, as well as concerns for iatrogenic injuries:

*‘But the more I thought about it, the more um the more I started reading into the iatrogenic injuries that happen because you know there’s this practice if baby need resuscitation, the guidelines that you cut and clamp immediately. And I really began to be quite concerned about that, everything I could get my hands on in terms of papers, on this, the evidence was saying you need to resuscitate with the cord intact*.’ (Jenny, int, In: 27–27).

The women’s research led them to educate themselves (and often their partners) about emergency scenarios such as: shoulder dystocia, haemorrhage, cord prolapse and the baby needing resuscitation, all of which they felt that they could manage themselves whilst awaiting emergency services:

*‘Yes, it is seen that if you freebirth, you would stay at home regardless and that you know you don't have anything else in place. I gave my husband a few things to read so in an emergency he would know what to do while we waited for help.‘*(Jane, int, In: 73–73).

Therefore, as the midwives role was to *‘spot signs of morbidity’,* a role they felt capable of doing themselves, it was felt that the *‘midwives couldn’t do anything to make it a better birth’*.

Most women (particularly for those who were freebirthing for the first-time) sought assurance of their low-risk status via antenatal midwifery checks, and decided that as long as the pregnancy remained *‘normal’* they would *‘stay home’* [freebirth]. As their pregnancies were categorised as ‘low risk’ the women believed that the possibility of complications was unlikely. This process of engagement and disengagement demonstrated how the women valued affirmation of clinical wellbeing during pregnancy to support their decision to freebirth. The women’s engagement with midwives during the antenatal period lessened with subsequent freebirths. The women reported they would access antenatal care on an adhoc basis, and some preferred to do their own antenatal checks such as blood pressure and urinalysis. These women made evident a pro-active approach which shifted the responsibility of birthing their babies away from maternity professionals and back to themselves:

*‘Which is usually people’s first reactions, to tell you that it is very dangerous. When actually you are the one who has got the most interest in the baby being ok. So I find it quite, quite uh, ironic, how medical staff or relatives or friends telling women who plan to freebirth that they shouldn’t, that it is something that shouldn’t be doing, when who has got the most interest in the baby being ok? It’s the mother and then the father.’* (Jenny, int, In: 68–68).

## Discussion

This study has identified key factors that influenced women’s decision to freebirth in a UK context. The women framed their decision making through the accounts of their personal circumstances and previous life experiences demonstrating that for them, these external influences were pertinent to the decision making process. With the exception of one participant, the women were making an active choice based upon their previous birth experiences. For many their decision was borne out of a previous negative birth experience. For three of the women, further negative experiences with maternity services (such as when making attempts to book a homebirth) served to be the deciding factor to freebirth. For these women, they lost faith in the maternity services to provide them with the care that was adequate for their needs. Therefore, in order to feel safe they opted to freebirth. For others this decision was borne out of a positive experience where women wanted to enhance their birthing experience and a midwife was considered redundant. The women supported and validated their freebirth decision through extensive research, accessing wider support, and a perception of risk which prioritised an undisturbed environment with only known and trusted people present.

The findings of the current study are supported by a number of those identified in the recent metasynthesis [13]. For example, women’s freebirth decision was influenced by former birth experience(s) that led to a rejection of any form of maternity care; they demonstrated ‘agency’ and ‘autonomy’ in terms of making childbirth decisions that were outside of socio-cultural norms and women displayed ‘faith in the birth process’ whereby they perceived that routine practices would interfere with normal birth processes. The impact of a previous birth experience(s) and poor quality care on future decision making is also reflected in the wider literature. For example, previous research highlights how women choose elective caesareans due to a previous poor experience [32] as well as how women opt for a homebirth following a traumatic caesarean birth [33] or negative hospital birth experience [34]. These insights also support the conclusions of a recent metasynthesis into the psychosocial impact of a traumatic birth in that there is little research into how health professionals should identify or respond to women who have experienced previous adversity [35]. Furthermore, they contribute to the growing evidence base of how abusive and disrespectful care from maternity professionals can lead to post-natal dysphoria [35, 36]. New insights generated by this study that were not explicit in the metasynthesis [13] include how past experiences influence decision making, the impact of health literacy and the impact of a limited homebirth service.

This study found that women’s life experiences were pertinent to their decision making process. Previous research has not identified or addressed the impact of how past events and wider life experiences can impact on birth decision making [37–39]. This understanding is crucial for maternity professionals, because in order to provide individualised and holistic care, midwives need to understand how women’s life ‘herstories’ may influence or impact on their needs, childbirth choices as well as their interactions with maternity professionals. Whilst individualised care is enshrined in the UK midwives code of conduct [40], in reality, time constraints, short staffing and busy workloads mean that this is not always possible [41–43].

Women demonstrated, which may well be reflective of their high level of educational attainment, that they were able to process complex technical information and make informed decisions pertinent to their circumstances. This is known as health literacy and is defined as *‘the ability to read, understand, evaluate and use health information to make appropriate decisions about health and health care’* ([34], p.4). Research by Smith et al. [39] demonstrated that people with greater health literacy are more likely to share responsibility for decision making, due to a need to retain ownership and control over their care decisions and to minimise unnecessary interventions. The women in this study went a step further and assumed all the responsibility for their childbirth experience. Furthermore, these findings from this study echoes that of Smith et al. [39] in which those with higher health literacy were more likely to independently seek knowledge away from the medical professionals by doing their own research.

A number of the women made a decision to freebirth due to negative interactions with professionals about their planned homebirth. This finding confirms those by Viisainen [44] who reported that women opting for community birth can face moralistic opposition, accusations of irresponsibility or receive conflicting advice about the safety of homebirths. It also concurs with a recent UK survey who found that 25 % of respondents would consider freebirthing when options for homebirth services were restricted [45]. Women in this study also expressed concern about *‘fearful’* midwives attending their homebirth which in turn reduced their feelings of safety. This presents as a cause for concern as the safety of homebirths in appropriately selected women has long been confirmed [46]. The NCT reports that homebirth service provision is variable and influenced by staffing levels and information that women receive from health care professionals [47]. Findings from the Birthplace study [46, 48] agree that wide variations in service provision, staffing levels, organisational structures and midwifery retention have caused inequitable service provision. The RCM [49] surveyed 553 self-selecting midwives about their attitudes to homebirth. Whilst the majority were positive about homebirth they reported that barriers such as on call demands, a lack of support and negative attitudes by the obstetric team, current staffing levels and a lack of confidence impeded the service they could offer. It is unclear from the current study as to whether access to a birth centre would have affected the women’s decision making, as provision of birth centres is variable across the UK [47]. All of the women reported upon issues surrounding safety and risk challenging the current risk discourse within both the medical and midwifery model of care. For these women, they felt safer without midwife checks and procedures during labour as they considered such interference during the birth process increased the risk of morbidity or mortality. This is counter to the findings of global evidence that midwifery care can improve outcomes [50], and may reflect the increasing criticism and resistance to the over use of intervention in developed countries [50]. These insights also support those by feminist authors who argue that the medicalisation of childbirth is at the crux of the problems that maternity services face [33, 51, 52]*.*

### Strengths and limitations

This is the first study of its kind, thereby offering a unique perspective as to why women choose to freebirth in the UK. The fact that the 10 participants had had a collective total of 33 births, together with varying experiences of maternity services at times makes the findings seem conflicting or contradictory. However these prior experiences provided important insights into why and how maternity services can influence women’s birthing decisions. A strength of this study concerns triangulation. A range of methods i.e. narratives, interviews and member checking were used to confirm and authenticate the findings. While the sample size for this type study is considered adequate [26], it would be beneficial to capture more participant’s views from different socio-economic and cultural backgrounds. The participants were self-selecting due to the nature of the recruitment process, therefore, the perspectives of participants who were not active online may have been missed. All of the participants were also of similar socioeconomic class which may not be fully reflective of all women that choose to freebirth. In all research there is the potential to bias the interpretations, therefore a future study could use several independent researchers to add strength to the interpretations.

## Conclusion

Complex and varied factors that encompass previous life events and birth experiences lead women to freebirth. The findings suggest that the UK based midwifery philosophy of woman-centred care that tailors care to individual needs is not always carried out, leaving women to feel disillusioned, unsafe and opting out of any form of professionalised care for their births. Maternity services need to provide support for women who have experienced a previous traumatic birth and for women and midwives to work together to restore relationships, and co-create birth plans that enable women to be active agents in their birthing decisions even if they challenge normative practices. The aim of a recent maternity services review by NHS England (56) is to improve maternity services for all. The fact that women choose to freebirth in order to create a calm, quiet birthing space that is free from clinical interruptions and that enhances the physiology of labour, should be a key consideration.

### Availability of data and materials

The dataset is available upon request.
